# MBP-Positive and CD11c-Positive Cells Are Associated with Different Phenotypes of Korean Patients with Non-Asthmatic Chronic Rhinosinusitis

**DOI:** 10.1371/journal.pone.0111352

**Published:** 2014-10-31

**Authors:** Dong-Kyu Kim, Min-Hyun Park, Dong-Yeop Chang, Kyung Mi Eun, Hyun-Woo Shin, Ji-Hun Mo, Eui-Cheol Shin, Hong Ryul Jin, Sue Shin, Eun Youn Roh, Doo Hee Han, Dae Woo Kim

**Affiliations:** 1 Department of Otorhinolaryngology-Head and Neck Surgery, Chuncheon Sacred Heart Hospital, Hallym University College of Medicine, Chuncheon, Korea; 2 Department of Otorhinolaryngology-Head and Neck Surgery, Boramae Medical Center, Seoul National University College of Medicine, Seoul, Korea; 3 Laboratory of Immunology and Infectious Diseases, Graduate School of Medical Science and Engineering, Korea Advanced Institute of Science and Technology, Daejeon, Korea; 4 Department of Pharmacology and Biomedical Science, Ischemic/Hypoxic Disease Institute, Seoul National University College of Medicine, Seoul, Korea; 5 Department of Otorhinolaryngology, Dankook University College of Medicine, Chonan, Korea; 6 Department of Laboratory Medicine, Boramae Medical Center, Seoul National University College of Medicine, Seoul, Korea; 7 Department of Otorhinolaryngology-Head and Neck Surgery, Seoul National University College of Medicine, Seoul, Korea; UMR INSERM U866, France

## Abstract

**Background:**

Asthmatic nasal polyps primarily exhibit eosinophilic infiltration. However, the identities of the immune cells that infiltrate non-asthmatic nasal polyps remain unclear. Thus, we thought to investigate the distribution of innate immune cells and its clinical relevance in non-asthmatic chronic rhinosinusitis (CRS) in Korea.

**Methods:**

Tissues from uncinate process (UP) were obtained from controls (n = 18) and CRS without nasal polyps (CRSsNP, n = 45). Nasal polyps (NP) and UP were obtained from CRS with nasal polyps (CRSwNP, n = 56). The innate immune cells was evaluated by immunohistochemistry such as, eosinophil major basic protein (MBP), tryptase, CD68, CD163, CD11c, 2D7, human neutrophil elastase (HNE) and its distribution was analyzed according to clinical parameters.

**Results:**

In comparisons between UP from each group, CRSwNP had a higher number of MPB^+^, CD68^+^, and CD11c^+^ cells relative to CRSsNP. Comparisons between UP and NP from CRSwNP indicated that NP have a higher infiltrate of MBP^+^, CD163^+^, CD11c^+^, 2D7^+^ and HNE^+^ cells, whereas fewer CD68^+^ cells were found in NP. In addition, MBP^+^ and CD11c^+^ cells were increased from UP of CRSsNP, to UP of CRSwNP, and to NP of CRSwNP. Moreover, in UP from CRSwNP, the number of MBP^+^ and CD11c^+^ cells positively correlated with CT scores. In the analysis of CRSwNP phenotype, allergic eosinophilic polyps had a higher number of MBP^+^, tryptase^+^, CD11c^+^, 2D7^+^ cells than others, whereas allergic non-eosinophilic polyps showed mainly infiltration of HNE^+^ and 2D7^+^ cells.

**Conclusions:**

The infiltration of MBP^+^ and CD11c^+^ innate immune cells show a significant association with phenotype and disease extent of CRS and allergic status also may influences cellular phenotype in non-asthmatic CRSwNP in Korea.

## Introduction

Chronic rhinosinusitis (CRS) is one of the most common chronic inflammatory diseases of the nasal and paranasal sinuses. CRS is characterized by an accumulation of inflammatory cells and marked tissue remodeling [1]. Currently, CRS is divided into two types: CRS with nasal polyps (CRSwNP) and CRS without nasal polyps (CRSsNP) [2], [3] In the past several decades, numerous studies have been performed to investigate nasal polypogenesis in patients from Western countries. These studies suggest that CRSwNP in Western countries is characterized by a Th2-based immune response with abundant eosinophilic infiltration, high levels of Interleukin (IL-5), and low levels of TGF-β. In contrast, CRSsNP shows a predominantly Th1-based immune response with elevated expression of TGF-β [3]–[5]. However, few studies have been conducted in Asian patients with CRS [6]–[11].

Studies that examined CRSwNP predominantly in Western patients show that eosinophilic inflammation was dominant in more than 80% of patients. Asthma was also present in nearly 40% of Western patients with CRSwNP [2]–[4]. In contrast to the findings in Western patients, several studies of CRSwNP in Asian patients describe that more than 50% of patients with CRSwNP demonstrated non-eosinophilic inflammation and also most patients did not have asthma [6]–[11]. Moreover, most studies demonstrate the distinct pathophysiologic features, including inflammatory T cell activation profile, remodeling pattern, and treatment course between eosinophilic CRSwNP and non-eosinophilic CRSwNP [10]–[13]. Nevertheless, the development of different types of CRS remains unclear.

Recent some studies describe an important role of the innate immunity on the initiation of adaptive immunity and thus, it seems to play an important role in pathogenesis of CRS [14]–[16]. Specifically, the type 2 innate lymphoid cells which produce IL-5 and IL-13 are responded to exposure of innate cytokines such as IL-25 and IL-33 [14]. In addition, these innate cytokines are produced by innate immune cells including, eosinophils, mast cell, macrophages, dendrite cells, basophils, and neutrophils [15]–[18]. However, to date, the relationship between innate immune cells and pathogenesis of CRS in Asian patients has not yet been investigated. Therefore, the main objective of this study was (1) to evaluate the distribution of innate immune cells and (2) to investigate any differences of its clinical relevance according to the CRS phenotype in non-asthmatic Korean patients with CRS.

## Materials and Methods

### Subjects

Sinonasal and polyp tissues were obtained from patients with CRS during routine functional endoscopic sinus surgery. The diagnosis of CRS was based on personal history, physical examination, nasal endoscopy, and findings from computed tomography (CT) scans of the sinuses according to the Sinus and Allergy Health Partnership (1). Exclusion criteria were as follows: (1) younger than 18 years of age, (2) asthmatic or sensitive to aspirin, (3) prior treatment with antibiotics, systemic or topical corticosteroids, or other immune-modulating drugs for 4 weeks before surgery, (4) unilateral rhinosinusitis, antrochoanal polyp, allergic fungal sinusitis, cystic fibrosis, or immotile ciliary disease. Control tissues were obtained from patients without any sinus diseases during rhinologic surgeries, such as skull-base surgery, lacrimal-duct surgery, and orbital-decompression surgery. In this study, we obtained UP tissues from control subjects and inflamed UP tissues from the patients with CRS, including CRSsNP and CRSwNP. We also evaluated NP tissues from patients with CRSwNP. Each sample was divided into two parts. One was fixed in 10% formaldehyde and embedded in paraffin for histological analysis; the other was immediately frozen at −80°C for future mRNA and protein isolation. All patients provided written informed consent. This study was approved by the internal review board of Seoul National University, Boramae Medical Center. The severity of sinus disease was determined with the Lund-MacKay scoring system (0–24) [19]. Polyps were graded by size and extent in both nasal cavities on a scale of 0 to 6.^1^ Postoperative endoscopy findings were scored at 6 months after surgery according to the Lund-Kennedy methodology, which ranges from 0 to 20 [20]. The atopic status was determined by the ImmunoCAP assay (Phadia, Uppsala, Sweden), which detects IgE antibodies against six common aeroallergens (house dust mite mixture, tree mixture, weed mixture, grass mixture, animal dander mixture, and fungus mixture). The diagnoses of asthma and aspirin sensitivity were determined by an allergist based on lung function and challenge tests as applicable. Demographic and clinical characteristics of subjects enrolled in the study are shown in Table 1.

**Table 1:** Patient characteristics and type of method.

	Control	CRSsNP	CRSwNP	
Total no. of subjects	N = 18 (5 male)	N = 45 (23 male)	N = 56 (35 male)	
Tissue used	UP	UP	UP	NP
Age (yr), mean (SD)	41 (19)	49 (12)	49 (13)	46 (16)
Allergic rhinitis, N (%)	0 (0%)	16 (35%)	7 (36%)	16 (37%)
Asthma, N	0	0	0	0
Aspirin sensitivity, N	0	0	0	0
Lund-Mackay CT score	0 (0)	8.8 (5.0)	14.0 (6.5)	15.2 (6.6)
Lund-Kennedy score	NA	0.9 (1.6)	3.2 (2.5)	3.4 (2.8)
Blood eosinophil (%)	3.6 (2.3)	5.4 (3.8)	4.1 (3.6)	3.6 (3.4)
Polyp characteristics (Eosinophilic/Non-eosinophilic)	NA	NA	NA	22/34
**Methodologies used**				
Tissue IHC	18	45	56	56
FACS	0	10	0	11
Tissue mRNA	18	25	19	25
Tissue homogenates	7	13	14	30

Lund-Kennedy score was evaluated at postoperative 6 months.

### Definition of polyps: eosinophilic vs. non-eosinophilic; allergic vs. non-allergic

Paraffin-embedded tissue sections (5 µm) were stained with hematoxylin and eosin. Two physicians who were blinded to the diagnostic and clinical data independently evaluated the slides. The number of eosinophils was counted at a high-power field (400×), in which eosinophils in the mucosa were the densest cellular infiltrate beneath the epithelium. Five visual fields were examined per section to determine the mean percentage of inflammatory cells that were eosinophils. In this study, CRSwNP were classified as eosinophilic if eosinophils comprised more than 10% of the inflammatory cell population, and as non-eosinophilic if eosinophils comprised less than 10% of the inflammatory cells [21]. Allergic CRSwNP were defined as NP tissues from patients who had allergic symptoms and positive results on the ImmunoCAP assay [22].

### Immunohistochemistry

Immunohistochemical staining was performed with polink-2, polymerized horseradish peroxidase (HRP), and broad DAB-Detection System (Golden Bridge International Labs., WA, USA). Briefly, after deparaffinization, sections were incubated in 3% hydrogen peroxidase to inhibit endogenous peroxidases. Heat-induced epitope retrieval was then performed by microwaving samples in 10 mmol/L citrate buffer (pH 6.0). The sections were incubated for 60 minutes (min) at room temperature in a primary antibody. The primary antibodies were mouse anti-human eosinophil major basic protein (MBP) (1∶50; Santa Cruz Biotech., California, USA), mouse anti-human CD11c (1∶5; BD Pharmingen, California, USA), mouse anti-mast-cell tryptase (1∶500; Abcam, Cambridge, UK), mouse anti-CD68 (1∶250; Abcam, Cambridge, UK), mouse anti-CD163 (1∶25; Abcam, Cambridge, UK), mouse anti-basophils (2D7) (1∶10; Abcam, Cambridge, UK), and anti-human neutrophil elastase (HNE) (1∶100; Abcam, Cambridge, UK). The sections were incubated in broad-antibody enhancer and polymer-HRP for rabbit and mouse antibodies. The sections were then stained with the DAB-Detection System. Finally, slides were counterstained with hematoxylin. The numbers of positive cells in the epithelium, glands, and submucosa were counted in the five densest visual fields (400×) by two independent observers, and the average values were determined.

### Quantitative real-time RT-PCR

We analyzed the mRNA expression levels of cytokine profiles (IL-5, IL-17A, IFN-γ, and TNF-α) and inflammatory markers (T-bet, GATA-3, and RORC) by real-time PCR. Total RNA was extracted from tissue samples with TRI reagent (Invitrogen, Carlsbad, CA, USA). One microgram of total RNA was reverse-transcribed to cDNA with a cDNA Synthesis Kit (amfiRivert Platinum cDNA Synthesis Master Mix, GenDEPOT). Quantitative real-time PCR was performed with a LightCycler 480 SYBR Green I Master (Roche, Mannheim, Germany). For analysis of IL-5 (Hs01548712_g1), IL-17A (Hs00174383_m1), IFN-γ (Hs00989291_m1), TNF-α (Hs01113624_g1), and GAPDH (Hs02758991_g1), pre-developed assay reagent (PDAR) kits of primers and probes were purchased from TaqMan assays (Life Technologies Korea, Seoul, Korea). In addition, the quantitative real-time PCR assay was performed with appropriate primers that specifically amplified T-bet, GATA-3, and RORC. Primers were as follows: T-bet, 5′-GTCAATTCCTTGGGGGAGAT-3′ for the forward primer and 5′-TCATGCTGACTGCTCGAAAC-3′ for the reverse primer; GATA-3, 5′-ACCACAACCACACTCTGGAGGA-3′ for the forward primer and 5′-TCGGTTTCTGGTCTGGATGCCT-3′ for the reverse primer; RORC, 5′-GCTGTGATCTTGCCCAGAACC-3′ for the forward primer and 5′-CTGCCCATCATTGCTGTTAATCC-3′ for the reverse primer; GAPDH, 5′-CATGGGTGTGAACCATGAGAA-3′ for the forward primer, 5′-GGTCATGAGTCCTTC CACGAT-3′ for the reverse primer. GAPDH was measured as a housekeeping gene for normalization. Cycling conditions were 95°C for 5 min, followed by 60 cycles at 95°C for 15 seconds (sec), 55°C for 20 sec, and 72°C for 20 sec. Data were analyzed with Sequence Detection Software version 1.9.1 (Applied Biosystems, Foster City, CA, USA). Relative gene expression was calculated by the comparative 2^-ΔΔCT^ method.

### Measurement of total and specific IgE

Total and of specific IgE to staphylococcal enterotoxins A (SEA), B (SEB), C (SEC) levels in nasal tissue homogenates were measured by using the ImmunoCAP assay according to the manufacturer's instructions. For preparation of tissue homogenates, harvested tissues were submersed in 1 mL phosphate-buffered saline (PBS) supplemented with 0.05% Tween-20 (Sigma-Aldrich, St Louis, MO) and 1% PIC (Sigma-Aldrich) per 0.1 g of tissue. And then, these tissues were homogenized with a mechanical homogenizer at 1,000 rpm for 5 min on ice. After homogenization, the suspensions were centrifuged at 3,000 rpm for 10 min at 4°C. The supernatants were separated and stored at −80°C until assay. The level of total IgE and antigen (SEA, SEB, SEC)-specific IgEs were measured using the CAP system.

### Flow cytometry

Nasal tissues, including NPs and ethmoidal mucosa, were gathered and minced into small pieces to isolate lymphocytes. The minced tissues were mechanically homogenized. The homogenates were filtered through a 70-*µ*m cell strainer (SPL Lifesciences, Pocheon, Korea). The filtered cells, including the nasal mucosal lymphocytes, were collected and cryopreserved until the time of analysis. The cryopreserved cells were thawed for analysis. At first, to exclude dead cells from analysis, cells were treated with the LIVE/DEAD Fixable Red Dead-Cell Stain Kit (Invitrogen, Carlsbad, CA, USA) according to the manufacturer's instructions. After washing, Cells were stained with BD horizon V500-conjugated anti-CD3 antibody (BD Biosciences, San Jose, CA, USA), APC-H7-conjugated anti-CD4 antibody (BD Biosciences), APC-conjugated anti-CCR4 antibody (R&D Systems, Minneapolis, MN, USA), and PE-conjugated anti-CXCR3 antibody (R&D Systems) in the dark for 30 min at 4°C to stain chemokine receptors on CD4^+^ T cells. Cells were washed and analyzed by multicolor flow cytometry. Flow cytometry was performed with an LSR II instrument (BD Biosciences), and data were analyzed with FlowJo software (Treestar, Ashland, OR, USA).

### Statistical analysis

Statistical analyses were performed with Graphpad Prism software 6.0 (GraphPad software Inc, La Jolla, CA) and SPSS 18.0 (SPSS, Inc, Chicago, Ill). Data were expressed in bar charts that represent medians and interquartile ranges. Statistical analyses were performed with the Kruskal-Wallis test and the Mann-Whitney U 2-tailed test for unpaired comparisons. Comparisons between groups were performed with the Kruskal-Wallis test to establish the significance of intergroup variability. Between-group comparisons were performed with the Mann-Whitney U test. Correlations were determined with the Spearman test. The significance level was set at an α-value of 0.05.

## Results

### Distribution of innate immune cell in different types of non-asthmatic CRS

**Figure 1:**
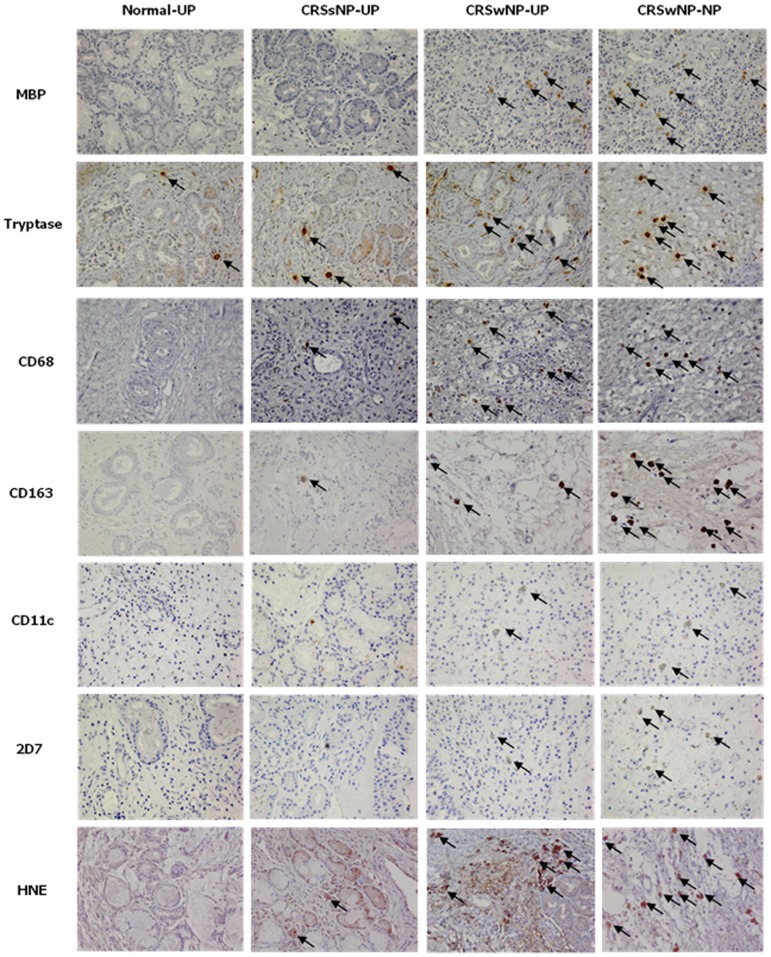
The immunohistochemical detection of innate immune cells was performed by using MBP, tryptase, CD68, CD163, CD11c, 2D7, and HNE (magnification x400). UP, uncinate process tissue; NP, nasal polyp tissue; CRSsNP, chronic rhinosinusitis without nasal polyps; CRSwNP, chronic rhinosinusitis with nasal polyps; MBP, anti-human eosinophil major basic protein; HNE, anti-human neutrophil elastase.

**Figure 2:**
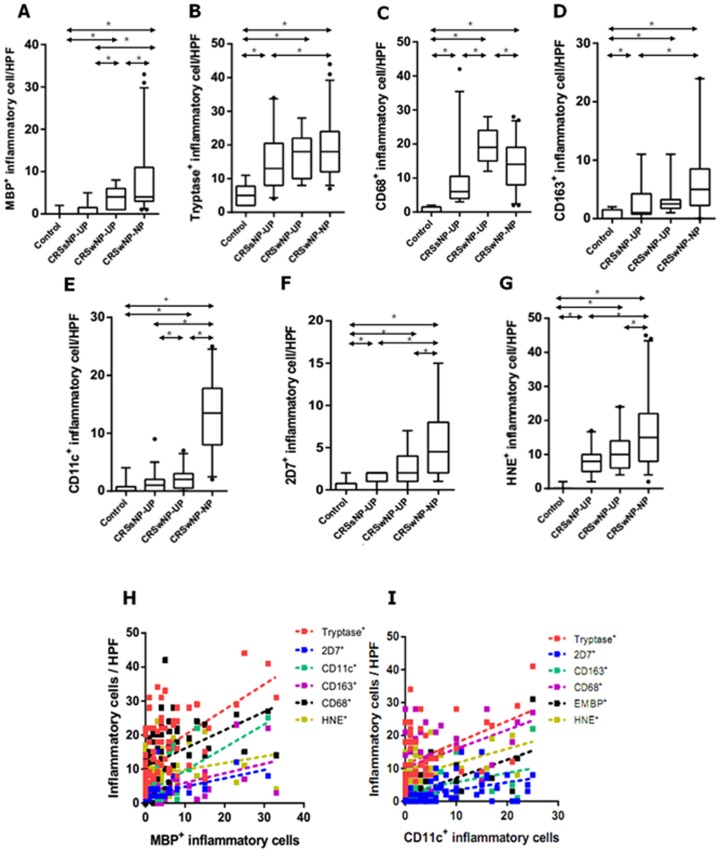
The number of MBP^+^ *(A)*, tryptase^+^ *(B)*, CD68^+^ *(C)*, CD163^+^ *(D)*, CD11c^+^ *(E)*, 2D7^+^ *(F)*, and HNE^+^ *(G)* cells in nasal mucosal tissues was compared according to different types of chronic rhinosinusitis. Correlation between innate immune cells was analyzed; in terms of MBP^+^ cell *(H)* and CD11c^+^ cell *(I)*. UP, uncinate process tissue; NP, nasal polyp tissue; CRSsNP, chronic rhinosinusitis without nasal polyps; CRSwNP, chronic rhinosinusitis with nasal polyps (*P<.05).

To investigate the cellular distribution of innate immune cells, we performed immunohistochemistry on UP tissues from controls, CRSsNP, and CRSwNP, and on NPs from CRSwNP (Fig. 1). Figure 2 shows the comparison of cellular distribution in nasal mucosal tissues according to different types of chronic rhinosinusitis. UP tissues from those with CRSsNP showed a significantly greater number of tryptase^+^ cells, CD68^+^ cells, CD163^+^ cells, 2D7^+^ cells, and HNE^+^ cells compared to those from the controls. In contrast, UP tissues from patients with CRSwNP had increased numbers of MBP^+^ cells, CD68^+^ cells, and CD11c^+^ cells compared to the UP tissues from the CRSsNP group. Examination of the cell populations in UP and NP tissues from the CRSwNP group showed that greater numbers of MBP^+^ cells, CD11c^+^ cells, 2D7^+^ cells, and HNE^+^ cells were present in NP tissues, whereas the number of CD68^+^ cells were decreased in NP tissues. In addition, the MBP^+^ and CD11c^+^ cells were increased from UP of CRSsNP, to UP of CRSwNP, and to NP of CRSwNP (Fig. 2A and 2E). On analyzing the relationship between the distribution of innate immune cells (Fig. 2H and 2I), we found that the number of MBP^+^ cells was strongly correlated with other immune cells (r = 0.417***, MBP vs. CD68; r = 0.618***, MBP vs. CD11c; r = 0.543***, MBP vs. tryptase; r = 0.557**, MBP vs. 2D7; r = 0.380*, MBP vs. CD163). In addition, the number of CD11c^+^ cells also showed strong correlation with several immune cells (r = 0.618***, CD11c vs. MBP; r = 0.357*, CD11c vs. tryptase; r = 0.490***, CD11c vs. 2D7; r = 0.427**, CD11c vs. CD163) (*P<.05, **P<.01, and ***P<.001).

### Correlation of innate immune cells and clinical implications in CRSwNP

The relationships between innate immune cells and clinical parameters of CRSwNP are shown in Table 2. Interestingly, in CRSwNP, the extent of disease was positively correlated with the number of MBP^+^ cells (r = 0.516, *P*<.05) and CD11c^+^ cells (r = 0.449, *P*<.05) in UPs from CRSwNP. Meanwhile, polyp grade and postoperative findings was not significantly correlated with all types of innate immune cells.

**Table 2:** Relationship between immunohistochemistry analysis and clinical parameters.

Tissue	Positive cell	Clinical parameters		
		CT score	Polyp grade	LK score at 6 months
**UP in CRSwNP**	MBP^+^	**0.516** *	0.371	0.471
	Tryptase^+^	0.228	0.204	0.066
	CD68^+^	0.128	−0.151	0.224
	CD163^+^	−0.044	−0.122	0.354
	CD11c^+^	**0.449** *	0.358	0.458
	2D7^+^	0.112	−0.120	0.125
	Elastase^+^	−0.192	−0.298	−0.259
				

LK score means Lund-Kennedy score.

*P<.05.

### Cellular pattern of innate immune cells according to clinicohistologic parameters in CRSwNP

**Figure 3:**
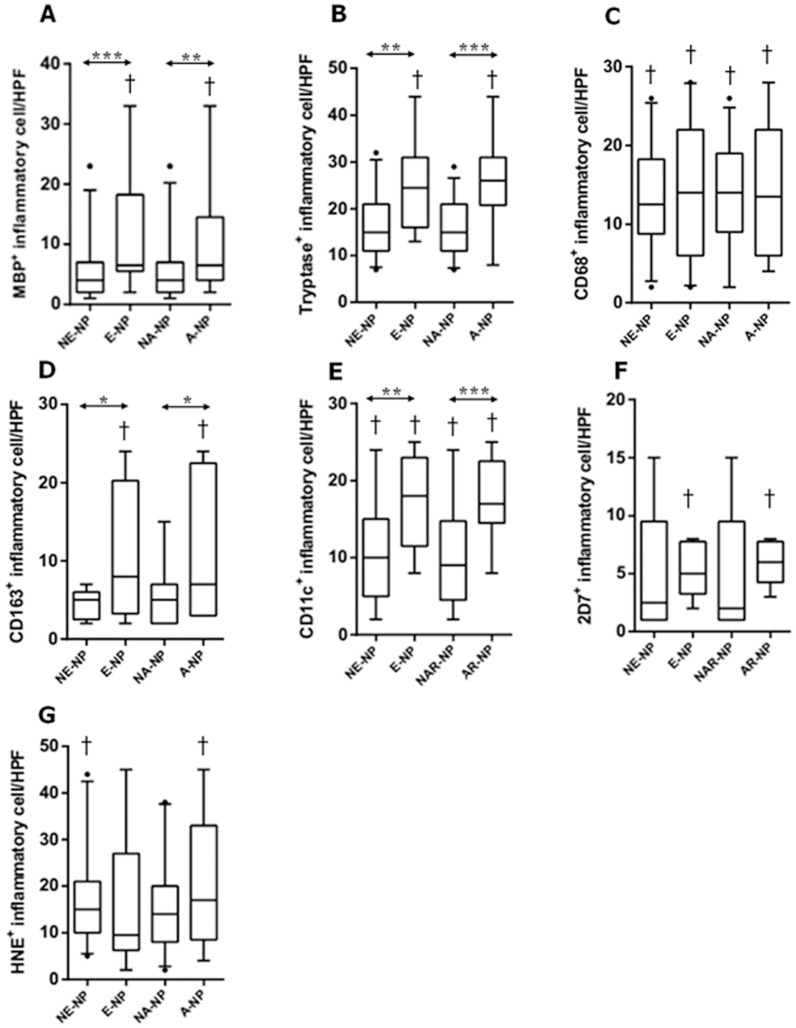
The number of MBP^+^ *(A)*, tryptase^+^ *(B)*, CD68^+^ *(C)*, CD163^+^ *(D)*, CD11c^+^ *(E)*, 2D7^+^ *(F)*, and HNE^+^ *(G)* cells in nasal mucosal tissues was compared according to clinicohistologic parameter: NE-NP, non-eosinophilic CRSwNP; E-NP, eosinophilic CRSwNP; NA-NP, non-allergic CRSwNP; A-NP, allergic CRSwNP (*P<.05, **P<.01, ***P<.001). †symbol means statistical significance when it compared with uncinate process tissue from CRSwNP.

**Figure 4:**
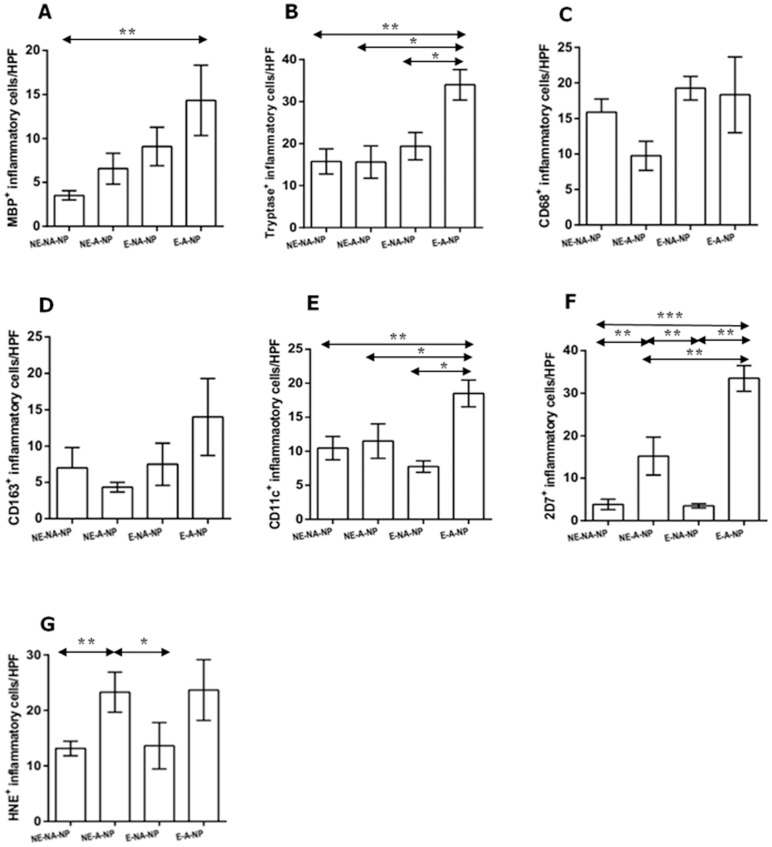
Comparison of MBP^+^ cell, tryptase^+^ cell, CD68^+^ cell, CD163^+^ cell, CD11c^+^ cell, 2D7^+^ cell, and HNE^+^ cell were performed in nasal polyp tissues among four groups: non-eosinophilic non-allergic (NE-NA-NP), non-eosinophilic allergic (NE-A-NP), eosinophilic allergic (E-NA-NP), eosinophilic allergic NP (E-A-NP) (*P<.05, **P<.01, ***P<.001).

We evaluated the distribution patterns of innate immune cells in different types of CRSwNP. As illustrated in Figure 3, there was a significant higher count of MBP^+^, tryptase^+^, CD163^+^, and CD11c^+^ cells in eosinophilic NP compared with non-eosinophilic NP. Compared with non-allergic NP, the numbers of MBP^+^, tryptase^+^, CD163^+^, and CD11c^+^ cells were also significantly increased in allergic NP. Meanwhile, in non-eosinophilic NP, overall innate immune cells were decreased compared with eosinophilic or allergic NP. Furthermore, in comparison between UP from CRSwNP and NP from non-eosinophilic NP, the number of HNE^+^ cells and CD11c^+^ cells were significantly higher in NP tissues and UP tissues. However, we found that less number of CD68^+^ cells was present in NP tissues. Based on the above results, we classified NP samples into four groups: non-eosinophilic non-allergic, non-eosinophilic allergic, eosinophilic non-allergic or eosinophilic allergic (Fig. 4). The frequency of MBP^+^ cell infiltration was higher in eosinophilic allergic NP than in non-eosinophilic non-allergic NP (Fig. 4A). Moreover, eosinophilic allergic NP had more tryptase^+^ cells, CD11c^+^ cells, and 2D7^+^ cells than observed in other types of CRSwNP (Fig. 4B, 4E, and 4F). In addition, compared with non-eosinophilic non-allergic NP and eosinophilic non-allergic NP, significantly increased numbers of 2D7^+^ cells and HNE^+^ cells were found in non-eosinophilic allergic NP (Fig. 4F and 4G). These data imply that allergic status influence cellular distribution in eosinophilic or non-eosinophilic NP.

### Levels of cytokine profiles, total IgE, and specific IgE to staphylococcal enterotoxins A, B, C in different types of non-asthmatic CRS

We evaluated the level of cytokine profiles in different types of CRS. Expression levels of IL-5, IL-17A, and IFN-γ were significantly increased in the UP tissues from patients with CRSwNP compared with those from controls (Shown in Figure S1A, B, and C). In addition, we found that IL-5 and IL-17A were up-regulated in the NP tissues from patients with CRSwNP compared to those from controls. On the analysis of TNF-α titer, there was no statistical difference among the UP tissues from different groups, but TNF-α levels were nearly significantly increased in UP tissues from patients with CRSwNP in comparison with controls (*P* = .056, *S1D*). To detect total and antigen-specific IgE (SEA, SEB, and SEC) levels, nasal tissue homogenates was examined by CAP system in different types of CRS. We found that NP tissues had significantly higher expression levels of total IgE in tissue homogenates than UP tissues from controls (Shown in Figure S1E). However, no significant difference in any type of specific IgE expression was observed among different study groups (Shown in Figure S1F, G, and H).

### T cell differentiation in different types of non-asthmatic CRS

We investigated the expression levels of mRNAs for T-bet, GATA-3, and RORC in UP tissues from non-asthmatic patients with CRSsNP, patients with CRSwNP, and controls. Interestingly, the mRNA levels of T-bet, GATA-3, and RORC were significantly up-regulated in CRSwNP compared with those in controls (*P* = .020, *P* = .029, *P* = .041) (Shown in Figure S2). Moreover, T-bet and RORC mRNA expression levels were significantly increased in CRSwNP compared with those in CRSsNP (*P* = .038, *P* = .030). However, there were no differences in the levels of T-bet, GATA-3, and RORC3 in the CRSsNP group and control group, although differences in T-bet expression approached significance (*P* = .065).

To confirm mixed T cell differentiation in non-asthmatic CRSwNP, CD4^+^ T cells was examined by flow-cytometric measurement of CXCR3 and CCR4 as markers of Th1 and Th2 activation (Shown in Figure S3). We found that all of the CRSsNP samples predominantly expressed CXCR3. In contrast, 9 out of 11 samples showed both CCR4 and CXCR3 expression of T cell in non-asthmatic nasal polyps. In detail, CRSwNP demonstrated enhanced levels of CCR4 in 4 of 11 samples, and others (7 samples) showed increased CXCR3 compared with CCR4. In the present study, flow-cytometric analysis confirmed that CRSsNP samples were characterized by Th1-based CD4^+^ T-cell differentiation, whereas CRSwNP demonstrated Th1/Th2- mixed T-cell differentiation.

## Discussion

In the present study, we evaluated the distribution of innate immune cells and its clinical impact in different type of CRS in non-asthmatic Korean patients. Several stains were conducted in order to compare the innate immune cells according to the phenotype of CRS: MBP for eosinophils, tryptase for mast cells, CD68 for M1 polarized macrophages, CD163 for M2 polarized macrophages, CD11c for dendritic cells, 2D7 for basophils, and HNE for neutrophils. In addition, it has been well-known that UP is one of the common sites in which NPs develop [1], [2]. Thus, we performed a comparative study between UPs or between UP and NP to detect the differences of innate immune cells during disease progression in non-asthmatic CRS patients. To our knowledge, this is the first study to investigate immunohistologic features of innate immunity according to the phenotype of CRS in non-asthmatic patients. Importantly, in the immunohistochemical study of UP tissues, we found that non-asthmatic patients with CRSwNP had a higher infiltrate of all kinds of innate immune cells in UP tissues than that of normal controls, whereas other cells except for MBP^+^ and CD11c^+^ cells were increased in UP tissues from CRSsNP compared to controls. In addition, CRSwNP showed more severe MBP^+^ cells, CD68^+^ cells, and CD11c^+^ cells infiltration than CRSsNP. This is consistent with evidence that various innate immune cells are associated with the pathogenesis of CRS [16], [22].

Until now, the relationship between innate immune cells and nasal polypogenesis has not yet been investigated. In this study, we evaluated the effector cells that considered to contribute for nasal polypogenesis using comparison of UP and NP from CRSwNP. We found that NP tissues showed excessive accumulation of MBP^+^ cells, CD11c^+^ cells, 2D7^+^ cells, and HNE^+^ cells compared to UP tissues from CRSsNP or CRSwNP. Although CD11c is not exclusively as marker for dendritic cell, these data are in agreement with previously reported elevated eosinophils, dendritic cells, basophils, and neutrophils in CRSwNP [18]–[22], [23]–[27]. In addition, in non-asthmatic CRS patients, the infiltration of MBP^+^ and CD11c^+^ cells increased gradually with the disease progression (from control to CRSsNP and to CRSsNP). Furthermore, we found a positive correlation between these and disease extent in UP tissues from CRSwNP. Thus, our results suggest that MBP^+^ cells and CD11c^+^ cells may be major effector cells for nasal polypogenesis in non-asthmatic Korean patients with CRSwNP.

Moreover, our study showed the difference macrophage infiltration according to the polarization such as M1 and M2. Generally, M1 macrophages are characterized by pro-inflammatory function and promotion of Th1 response, while M2 macrophages are immunosuppressive and have been related to the promotion of Th2 immune response [28]–[30]. In our study, CD68 for M1 marker and CD163 for M2 marker are used to identify macrophages in tissue sections. Interestingly, we found that the number of CD68^+^ cells was increased in UP tissues from control to CRSsNP and to CRSwNP, whereas CD68^+^ cells were decreased in NPs compared to UP tissues from CRSwNP. Meanwhile, the number of CD163^+^ cells was increased in UP tissues from CRS compared to controls, while the number of CD163^+^ cells in CRSwNP was not different between NPs and UP. These results suggest that, in addition to MBP^+^ and CD11c^+^ cells, macrophage polarization may also contribute to nasal polypogenesis in non-asthmatic Korean patients with CRS.

In comparison with non-eosinophilic NP and non-allergic NP, this study showed that eosinophilic NP and allergic NP showed significantly an increased numbers of MBP^+^, tryptase^+^, CD163^+^, and CD11c^+^ cells, respectively. Base on the above results, we analyzed cellular pattern according to the clinicohistologic parameter. Thus, we found that the number of MBP^+^, tryptase^+^, 2D7^+^, and CD11c^+^ cells was increased in eosinophilic allergic NP compared with non eosinophilic non allergic NP. Furthermore, the infiltration of 2D7^+^ and HNE^+^ cells was higher in non-eosinophilic allergic NP than in non-eosinophilic non-allergic NP and eosinophilic non-allergic NP. Considering that eosinophils and neutrophils are the main cellular components of eosinophilic NP and non-eosinophilic NP, respectively [3]–[5], [8], [10], [31], we hypothesize that allergic conditions may play an important role in determining the cellular phenotype of nasal polyps in non-asthmatic Korean patients with CRSwNP. Thus far, some studies suggest that nasal polypogenesis might be related with allergy [6], [12], [32], [33]. Other reports indicate that allergic status may not significantly affect inflammatory mediators in nasal polyps [3], [34], [35]. Therefore, the role of allergic status in nasal polypogenesis remains unclear. However, we showed for the first time that the distribution of innate immune cells was remarkably different depending on the allergic status of non-asthmatic patients with CRSwNP. These findings may provide new insight into the mechanism of pathogenesis in non-asthmatic patients with CRSwNP.

Comparing with Western studies, data from previous Asian reports show some differences in the inflammatory features and remodeling pattern. Some studies from southern Chinese patients discovered that CRSwNP were characterized by neutrophil-dominant inflammation and a significant increase in Th1/Th17-cytokines [7]–[9]. Other study based on Asian patients found that more than 50% of cases of CRSwNP exhibited non-eosinophilic inflammation with milder Th1/Th2/Th17-mixed responses [10]. Study for remodeling in Asian patients with CRS described that hypoxic condition may induce neutrophilic inflammation with increase of TGF-b2 in non-eosinophilic CRSwNP and CRSsNP [11]. In this study, we also found that the expression level of IL-5, IL-17A, and IFN-γ mRNA in UP tissues was increased from CRSwNP compared with controls, whereas there was no statistical difference in the titer of SEA, SEB, and SEC among different types of CRS. Thus, to further investigate the phenotype of non-asthmatic Korean nasal polyps, we analyzed the mRNA expression of transcription factors, including T-bet, GATA-3, and RORC in UP tissues from CRS patients. In this study, the expression levels of T-bet, RORC and GATA-3 mRNAs were up-regulated in the CRSwNP group compared with the control groups. These data suggested that non-asthmatic Korean patients with CRSwNP have mixed Th1/Th2/Th17 responses, which is consistent with outcomes from Asian studies [7]–[10]. Moreover, to confirm our results regarding the expression levels of mRNA transcription factors, we conducted flow cytometry to examine chemokine receptors as typical cell markers for Th1 and Th2. CXCR3 and CCR5 are preferentially expressed on Th1 cells, whereas Th2 cells favor the expression of CCR3 and CCR4 [36], [37]. Analysis of T-cell expression in cells derived from nasal tissues revealed increased expression of Th1 in CRSsNP, whereas mixed expression of Th1/Th2 was observed in CRSwNP. Our flow cytometry results were consistent with our analysis of transcription factors, confirming mixed T-cell differentiation.

## Conclusions

In the present study, Korean nasal polyps were characterized by mixed T cell differentiation. Nevertheless, innate immune cells showing big changes between CRS phenotype are MBP^+^ and CD11c^+^ cells. In addition, MBP^+^ and CD11c^+^ cells were significantly associated with the disease extent of CRSwNP such as CT scores. Furthermore, our results demonstrate that allergic status may influence the cellular phenotype of innate immune cells in NPs from non-asthmatic Korean patients with CRSwNP.

## Supporting Information

Figure S1 **The expression levels of cytokines and total/antigen-specific IgE in nasal tissues homogenates were assessed by qRT-PCR and CAP system, respectively.** The mRNA expression levels of (A) IL-5, (B) IL-17A, (C) IFN-γ and (D) TNF-α are shown relative to the expression level of the GAPDH housekeeping gene. (E) Total IgE, (F) specific IgE to staphylococcal enterotoxins A [SEA], (G) specific IgE to staphylococcal enterotoxins B [SEB], and (H) specific IgE to staphylococcal enterotoxins C [SEC] levels in tissue homogenates were measured by CAP system (*P<.05, **P<.010, ***P<.001).
(TIF)

Figure S2 **The mRNA expression levels of T-bet, GATA-3, and RORC in UP from control, CRSsNP, and CRSwNP was measured by using real-time PCR.** UP, uncinate process tissue; CRSsNP, chronic rhinosinusitis without nasal polyps; CRSwNP, chronic rhinosinusitis with nasal polyps (*P<.05, ***P<.001).
(TIF)

Figure S3 **Different CD4^+^ T cell in EM from CRSsNP and NP from CRSwNP was detected by flow-cytometry using CXCR3 and CCR.** EM, ethmoidal mucosa; CRSsNP, chronic rhinosinusitis without nasal polyps; CRSwNP, chronic rhinosinusitis with nasal polyps (*P<.05, ***P<.001).
(TIF)
