# Supplementary figures and images for: MBP-Positive and CD11c-Positive Cells Are Associated with Different Phenotypes of Korean Patients with Non-Asthmatic Chronic Rhinosinusitis
Source: PLoS One. 2014 Oct 31;9(10):e111352. doi: 10.1371/journal.pone.0111352 (PMC4216068; doi:10.1371/journal.pone.0111352)

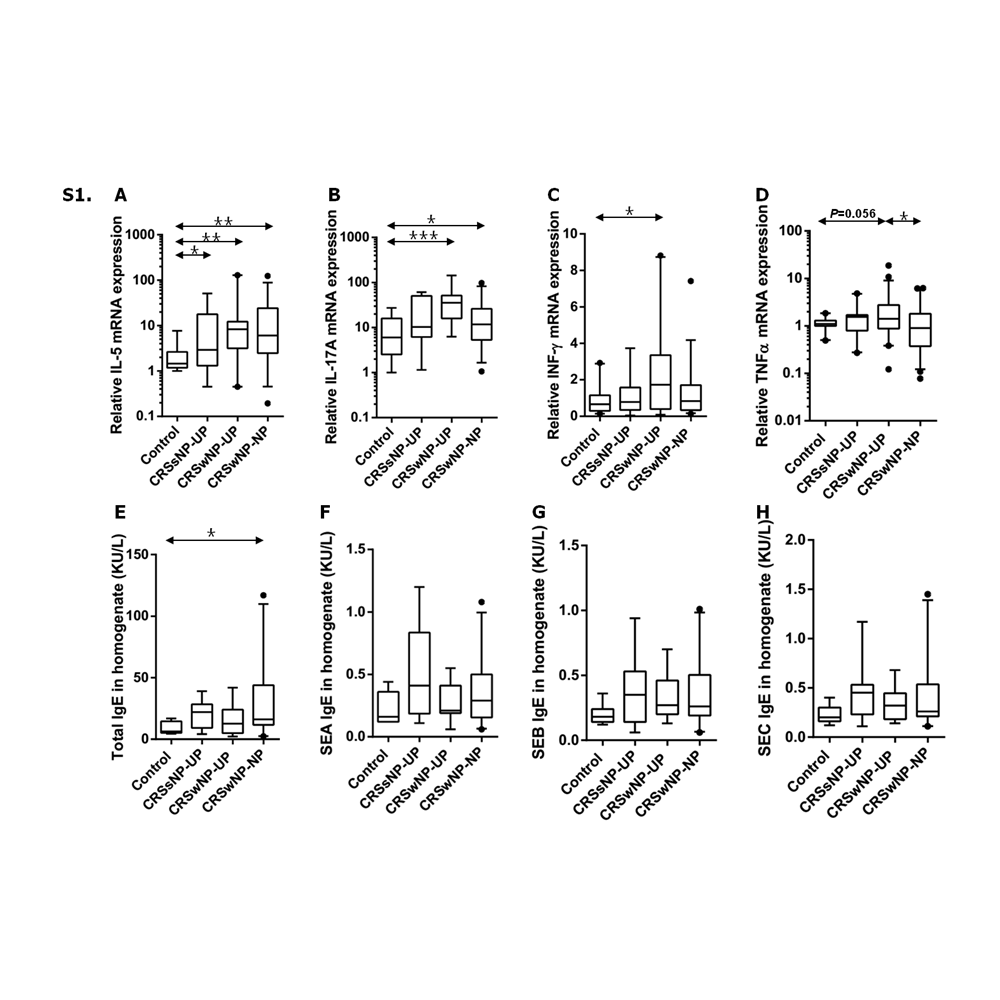

Supplement: Figure S1 — The expression levels of cytokines and total/antigen-specific IgE in nasal tissues homogenates were assessed by qRT-PCR and CAP system, respectively. The mRNA expression levels of (A) IL-5, (B) IL-17A, (C) IFN-γ and (D) TNF-α are shown relative to the expression level of the GAPDH housekeeping gene. (E) Total IgE, (F) specific IgE to staphylococcal enterotoxins A [SEA], (G) specific IgE to staphylococcal enterotoxins B [SEB], and (H) specific IgE to staphylococcal enterotoxins C [SEC] levels in tissue homogenates were measured by CAP system (*P<.05, **P<.010, ***P<.001). (TIF) [file pone.0111352.s001.tif]

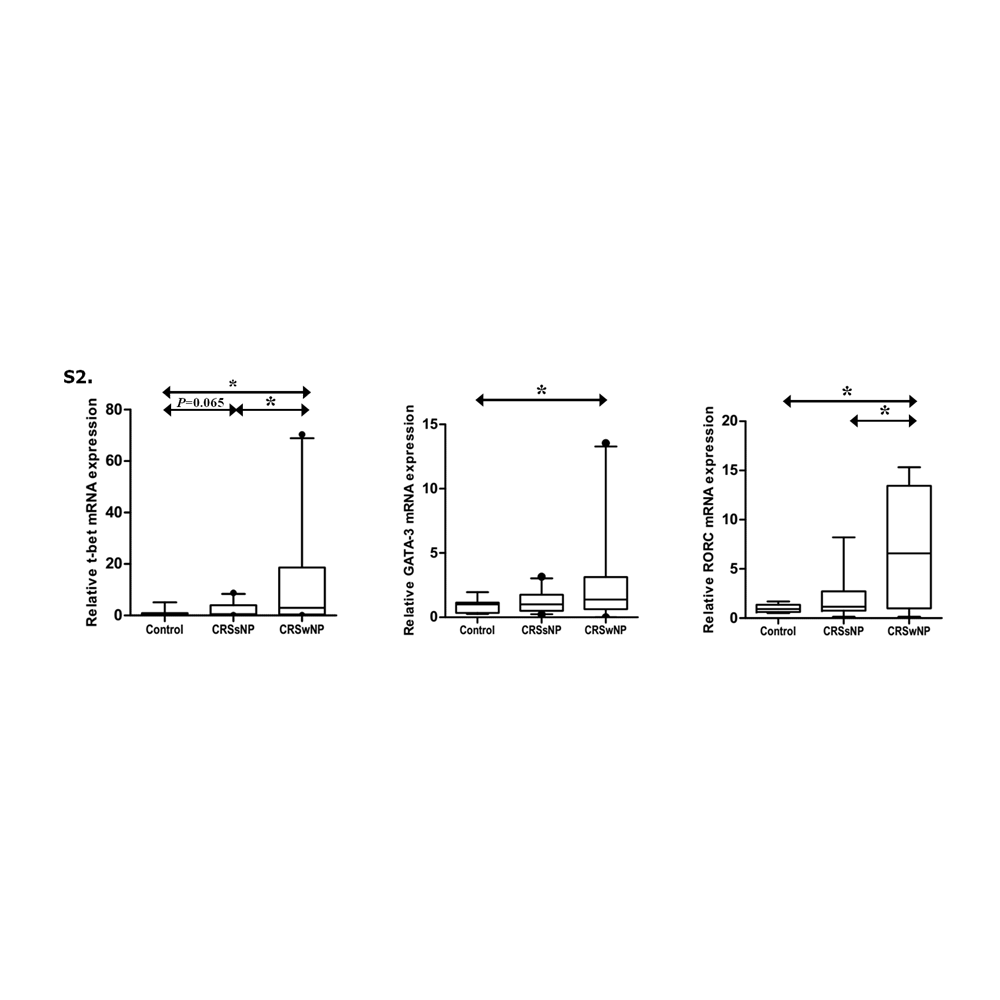

Supplement: Figure S2 — The mRNA expression levels of T-bet, GATA-3, and RORC in UP from control, CRSsNP, and CRSwNP was measured by using real-time PCR. UP, uncinate process tissue; CRSsNP, chronic rhinosinusitis without nasal polyps; CRSwNP, chronic rhinosinusitis with nasal polyps (*P<.05, ***P<.001). (TIF) [file pone.0111352.s002.tif]

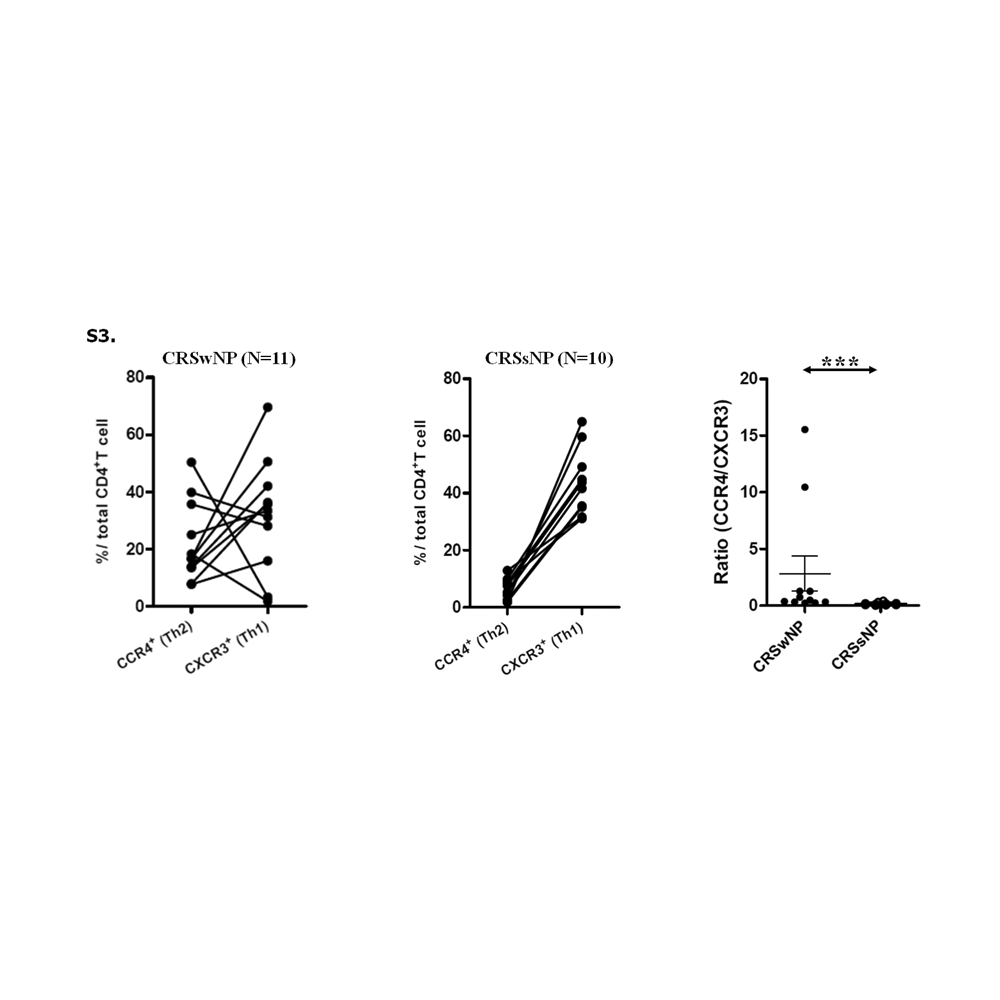

Supplement: Figure S3 — Different CD4+ T cell in EM from CRSsNP and NP from CRSwNP was detected by flow-cytometry using CXCR3 and CCR. EM, ethmoidal mucosa; CRSsNP, chronic rhinosinusitis without nasal polyps; CRSwNP, chronic rhinosinusitis with nasal polyps (*P<.05, ***P<.001). (TIF) [file pone.0111352.s003.tif]
